# A Comparative Assessment of the Chronic Effects of Micro- and Nano-Plastics on the Physiology of the Mediterranean Mussel *Mytilus galloprovincialis*

**DOI:** 10.3390/nano11030649

**Published:** 2021-03-07

**Authors:** Marco Capolupo, Paola Valbonesi, Elena Fabbri

**Affiliations:** Department of Biological, Geological, and Environmental Sciences, University of Bologna, Via Sant’ Alberto 163, 48123 Ravenna, Italy; paola.valbonesi@unibo.it (P.V.); elena.fabbri@unibo.it (E.F.)

**Keywords:** polystyrene, nano-fragmentation, marine bivalves, biomarkers

## Abstract

The ocean contamination caused by micro- and nano-sized plastics is a matter of increasing concern regarding their potential effects on marine organisms. This study compared the effects of a 21-day exposure to 1.5, 15, and 150 ng/L of polystyrene microplastics (PS-MP, 3-µm) and nanoplastics (PS-NP, 50-nm) on a suite of biomarkers measured in the Mediterranean mussel *Mytilus galloprovincialis*. Endpoints encompassed immunological/lysosomal responses, oxidative stress/detoxification parameters, and neurotoxicological markers. Compared to PS-MP, PS-NP induced higher effects on lysosomal parameters of general stress. Exposures to both particle sizes increased lipid peroxidation and catalase activity in gills; PS-NP elicited greater effects on the phase-II metabolism enzyme glutathione S-transferase and on lysozyme activity, while only PS-MP inhibited the hemocyte phagocytosis, suggesting a major role of PS particle size in modulating immunological/detoxification pathways. A decreased acetylcholinesterase activity was induced by PS-NP, indicating their potential to impair neurological functions in mussels. Biomarker data integration in the Mussel Expert System identified an overall greater health status alteration in mussels exposed to PS-NP compared to PS-MP. This study shows that increasing concentrations of nanoplastics may induce higher effects than microplastics on the mussel’s lysosomal, metabolic, and neurological functions, eventually resulting in a greater impact on their overall fitness.

## 1. Introduction

Plastic is the most prevalently used material in modern society; it supports nearly all socio-economic activities, including household, health, and food sectors, and its production has exponentially soared in the past 70 years, reaching 368 million tons in 2019 [1]. Around 40% of daily used plastics are represented by single-use items, such as bags, bottles, and/or packages, which can easily be subjected to improper use or disposal. The management of plastic waste has consequently emerged as a major environmental issue in the latest decades [2]. According to Pinto da Costa et al. [3], 79% of plastics sent to landfills finally find their way to oceans, for an estimated annual amount of 4.8–12.7 million tons. Plastic polymers do not tend to degrade in marine systems; rather, they experience a swift additive loss with consequent fragmentation into smaller pieces from the effect of weathering forces [2,4,5]. As a result, 94% of plastics currently present in oceans are represented by <5 mm fragments [3], conventionally referred to as microplastics (MP) [6].

Many recent investigations focused on the interaction between MP and the marine biota. Because of their small size, variable shape/color, and seawater-alike density, MP frequently ends up being accidentally ingested (directly or via biomagnification) by marine organisms from different trophic levels, causing adverse biological effects at different scales of observation [7,8]. The main documented alterations include gut occlusion and reduced feeding stimulus (associated with reduced growth/fertility) in turtles and seabirds [9,10], metabolic and reproductive disorders in fish [11,12], and behavioral/nutritional alterations in invertebrates, like arthropods, annelids, and mollusks [13,14,15]. Besides, recent studies on bivalves report the alterations of cellular, biochemical and ontogenetic pathways, accenting the risks posed by MP on the processes governing the physiological adaptation to external stressors and population recruitment patterns [16,17,18].

A recent paradigm in marine plastic pollution is that the progressive plastic fragmentation to the nanoscale will expose organisms to far more subtle effects than observed for micro-scaled fragments [4]. Plastics of a size < 100 nm, conventionally termed nanoplastics (NP) [19], may easily cross biological membranes, entering cells/tissue [20]; in addition, they display a relatively high surface to volume ratio, which may increase their reactivity toward organic compounds of endogenous or exogenous nature [4]. From a toxicological viewpoint, these features would enhance the NP’s potential to interfere with molecular, cellular, and/or physiological mechanisms, likely impacting higher biological functions in the long term. Exposure to NP was recently found to induce Reactive Oxygen Species (ROS) overproduction in the marine bacterium *Halomonas alkaliphila* [21], transcriptomic changes in the brine shrimp *Artemia franciscana* [22], and alterations of several biochemical/cellular pathways in mussels of the *Mytilus* spp., including lysosomal/biotransformation processes, ROS-scavenging mechanisms, immune response and gene expression [23,24,25]. Most of these studies focused on the acute or sub-chronic impact of virgin and/or conditioned (e.g., aminated or carboxilated) NP alone; on the other hand, little is still known about the differences between the effects of micro- and nano-sized plastics on the physiology of marine organisms, notably following prolonged exposures.

The aim of the present study is to comparatively assess the chronic effects of 3-µm polystyrene MP (PS-MP) and 50-nm polystyrene NP (PS-NP) on a suite of physiological, cellular, and biochemical parameters measured in the Mediterranean mussel *Mytilus galloprovincialis*. This species has been chosen based on its ease of collection/rearing, wide geographical distribution, filter-feeding habits, and well-known responsiveness to stress conditions [26]. A battery of biomarkers of environmental stress were measured following 21 days of mussel exposure to increasing concentrations of PS-MP and PS-NP.

## 2. Materials and Methods

### 2.1. Experimental Design

Sexually mature specimens of the Mediterranean mussel *Mytilus galloprovincialis* (4–6 cm length) were collected in a high-quality marine area (Cesenatico, NW-Adriatic Sea) and immediately transferred to laboratory, where they were acclimated for 5 days at 16 °C and 10:14 h (light:dark) photoperiod. Afterward, animals were divided into groups of 20 individuals and separately exposed for 21 days to 1.5, 15, and 150 ng/L of 3-µm PS-MP and 50-nm PS-NP (described in detail in SM, section S1) into tanks containing 20 L of filtered seawater (FSW), each representing the experimental replicates (N = 3). Treatments were selected with the aim to estimate the sub-lethal impact associated with the fragmentation of a fixed amount of plastics from the micro to the nanoscale, as is expected to occur in natural environments due to effects of weathering processes [4]. In terms of nominal concentration of particles/volume unit, chosen treatments corresponded to 100, 1000, and 10,000 PS-MP/L and 2.17 × 10^5^, 2.17 × 10^6^, and 2.17 × 10^7^ PS-NP/L. PS-MP and PS-NP concentrations were renewed daily after a water change. A control (CTR) treatment containing no particles was performed in parallel. After collection, mussel tissues were immediately analyzed for selected endpoints (hemolymph) or stored at −80 °C for subsequent analyses (gills, digestive gland). To debate the likelihood of biases due to inter-individual variability, data from each replicate (vessel) consisted of the mean (± Standard Error of Mean, SEM) level of selected endpoints from 4 randomly selected mussels per replicate (12 mussels per experimental condition). For biomarkers analyzed spectrophotometrically, data were normalized on the total protein content according to Lowry et al. [27].

### 2.2. Lysosomal Parameters

#### 2.2.1. Lysosomal Membrane Stability (LMS)

The effect of PS-MP and PS-NP on the lysosomal membrane stability (LMS) of mussel hemocytes was evaluated using the Neutral Red Retention Assay (NRRA) as described by Martinez-Gomez et al. [28]. The method is based on exposing hemocytes to a solution containing 0.01% Neutral Red (NR, Sigma-Aldrich ^®^, Milan, Italy) acidophile dye (which migrates into lysosomes) and on the assessment of the Neutral Red Retention Time (NRRT), which is the time (min) where more than 50% of the lysosomes released the dye into the cytosol.

#### 2.2.2. Histopathological Alterations

The lysosome to cytoplasm volume ratio (LYS/CYT) and the intra-lysosomal content of lipofuscin (LF) and unsaturated neutral lipids (NL) were assessed in histological cryo-sections of the mussel digestive gland obtained according to the UNEP/RAMOGE guidelines [29]. Glands of four mussels per tank were dissected, placed on aluminum supports for cryotomy (chuck), and rapidly frozen in N-hexane before being stored at −80 °C. Ten-µm gland sections were obtained using a cryotome at −30 °C, rapidly placed onto glass slides, and selectively stained for chosen parameters, as described below.

The LYS/CYT was performed according to Capolupo et al. [30] by selectively staining the reaction between the lysosomal hydrolase N-acetyl-β-hexosaminidase and a specific substrate. Briefly, sections were shaken for 20 min at 37 °C in the presence of the substrate naphtol AS-BI N-acetyl-β-D-glucosaminide (Sigma-Aldrich ^®^, Milan, Italy) and stained using the diazonium dye “Fast Violet B” (1 mg/mL in 0.1 M phosphate buffer, pH 7.4) (Sigma-Aldrich ^®^, Milan, Italy). The NL and LF content were assessed according to Martin-Diaz et al. [31]. For NL, sections were fixed in formol calcium for 15 min at 4 °C, transferred into 60% triethylphosphate (Carlo Erba ^®^, Milan, Italy) for 3 min. and stained in a 1% oil red O (Sigma-Aldrich ^®^, Milan, Italy) staining solution for 15 min in the dark. For LF, slides were fixed as for NL and selectively dyed for 5 min. in the dark using the Schmorl solution (1% ferric chloride and 1% potassium ferricyanide at a 3:1 ratio).

The three parameters were assessed at 40× magnification under a light microscope (Axioskop 40, Carl Zeiss, Milan, Italy) equipped with a digital camera (AxioCam MRc, Carl Zeiss, Milan, Italy). Four photos per gland were taken randomly and analyzed using the software Scion Image ver. 4.0.2.

### 2.3. Biochemical and Enzymatic Biomarkers

The content of malondialdehyde (MDA) and the activities of the enzymes glutathione S-transferase (GST), catalase (CAT), and acetylcholinesterase (AChE), were assessed spectrophotometrically in samples of mussel digestive gland and/or gills preliminarily processed at the specific conditions reported in SM (Table S1). The MDA content was quantified in digestive glands according to Franzellitti et al. [32] by interpolating the data of absorbance at 570 nm on a standard curve obtained using the MDA precursor 1,1,3,3-Tetramethoxypropan (TMOP, Sigma-Aldrich ^®^, Milan, Italy). GST and CAT activities were measured in both digestive glands and gills according to Capolupo et al. [33]. GST activity was evaluated at 340 nm by following the reaction kinetics between the enzyme and the substrate 1-chloro-2,4-dinitrobenzene (CDNB, Sigma-Aldrich ^®^, Milan, Italy) for 10 min (at 1 min intervals). Similarly, CAT activity was measured by tracking the decrease in the absorbance of samples at 240 nm for 2 min (at 10 s intervals) in the presence of 55 mM H_2_O_2_. AChE activity was determined in gills at 405 nm as described by Valbonesi et al. [34]. Samples were read at 405 nm for 10 min in the presence of 0.5 mM acetylthiocholine iodide and 0.33 mM 5.5′-dithiobis-2-nitrobenzoic acid (DTNB, Sigma-Aldrich ^®^, Milan, Italy).

### 2.4. Immunological Parameters

#### 2.4.1. Lysozyme Enzymatic Assay

The lysozyme-specific activity was assessed in the mussel hemolymph serum according to the protocol described by Chu and La Peyre [35]. The method is based on the decrease of absorbance of the hemolymph serum proportionally to the lytic action of lysozyme toward the bacterium *Micrococcus lysodeikticus* (Sigma-Aldrich ^®^, Milan, Italy). Firstly, 500 µL hemolymph was centrifuged at 4 °C for 10 min at 400× *g* to separate serum and hemocytes; hence, the supernatant (serum) was rapidly added to a solution containing 0.9 mg/mL of *M. lysodeikticus* cells in 0.066 M phosphate buffer (pH 6.24 at 25 °C) and read spectrophotometrically at 450 nm at 1 min intervals for 10 min total. The lysozyme activity was expressed as milliunits (mU)/mg of total proteins, 1U being the activity resulting in a decrease of 0.001 OD/mL sample.

#### 2.4.2. Phagocytosis

The phagocytic activity of mussel hemocytes has been assessed according to Canesi et al. [36]. Aliquots (20 µL) of hemolymph were placed and kept on microscope slides for 30 min at 16 °C in a dark and wet room to allow for hemocyte adhesion. Cells were exposed to a 0.05% solution of zymosan yeast (*Saccharomyces cerevisiae*, Sigma-Aldrich ^®^, Milan, Italy) particles (Sigma, Z4250), preliminarily stained with Neutral Red in 0.05 M Tris-HCl (2.5% NaCl, pH 7.6). After 1 h, hemocytes were fixed by adding Baker’s formol solution to each slide. The phagocytic efficiency was evaluated under a light microscope (Zeiss Axioskop 40, 40× magnification) and expressed as a percentage of hemocytes showing zymosan particles phagocytosis.

### 2.5. Statistical Analysis and Data Integration

Significant differences between treated and control mussels, as well as between PS-MP and PS-NP-treated mussels, were determined using the SIGMAPLOT 13 statistical package (Systat Software Inc. San Jose, CA, USA). After verifying the deviations from parametric Analysis of Variance (ANOVA) (normality: Shapiro–Wilk test; equal variance: Brown–Forsythe test), data were analyzed using a non-parametric Kruskal–Wallis followed by the Mann–Whitney (Rank Sum) U-test for multiple comparisons. In any case, the statistical difference was accepted when *p* < 0.05. A principal component analysis (PCA) was performed on data from single factors using the software PRIMER v6 (PRIMER-E Ltd., Albany, New Zealand) to ascertain the amount of variation explained by singles treatments/endpoints and allow for a comparable interpretation of the obtained results.

Biomarker data were integrated using the Mussel Expert System (MES) developed by Dagnino et al. [37] to define a synoptic health status index (HSI) for each tested condition. Data showing significant alterations were processed to obtain an alteration factor (AF), which is the ratio between the means of that specific value and control. An alteration level (AL) was generated by comparing any AFs with a specific threshold defined based on the toxicological profile expressed by each biomarker (i.e., increasing, decreasing, or bell-shaped response) and the hierarchical level at which alterations occur (cell, tissue, organism). Finally, the system applies a multiple-step algorithm to evaluate and define the A-E scaled HSI ranging from A (healthy) to E (pathological status) for each of the performed treatments. The most sensitive biomarker of the list was selected as a guide parameter (GP) and changes in its AL values were primarily considered by the system to interpret the overall mussel health status.

## 3. Results

A wide set of biological endpoints were measured in mussels exposed for 21 days to 1.5, 15, and 150 ng/L of PS-MP and PS-NP, including lysosomal biomarker of general stress (LMS, NL, and LYS/CYT), lipid peroxidation parameters (LF and MDA), antioxidant/detoxification enzymes (GST and CAT activities), neurological (AChE activity) and immunological parameters (lysozyme activity and phagocythosis). Finally, biological effects induced by the two particle types were integrated using the Mussel Expert System to identify the overall impact induced by their administration on the mussel health status.

### 3.1. Effects of PS-MP and PS-NP on Lysosomal Parameters of General Stress

**Figure 1:**
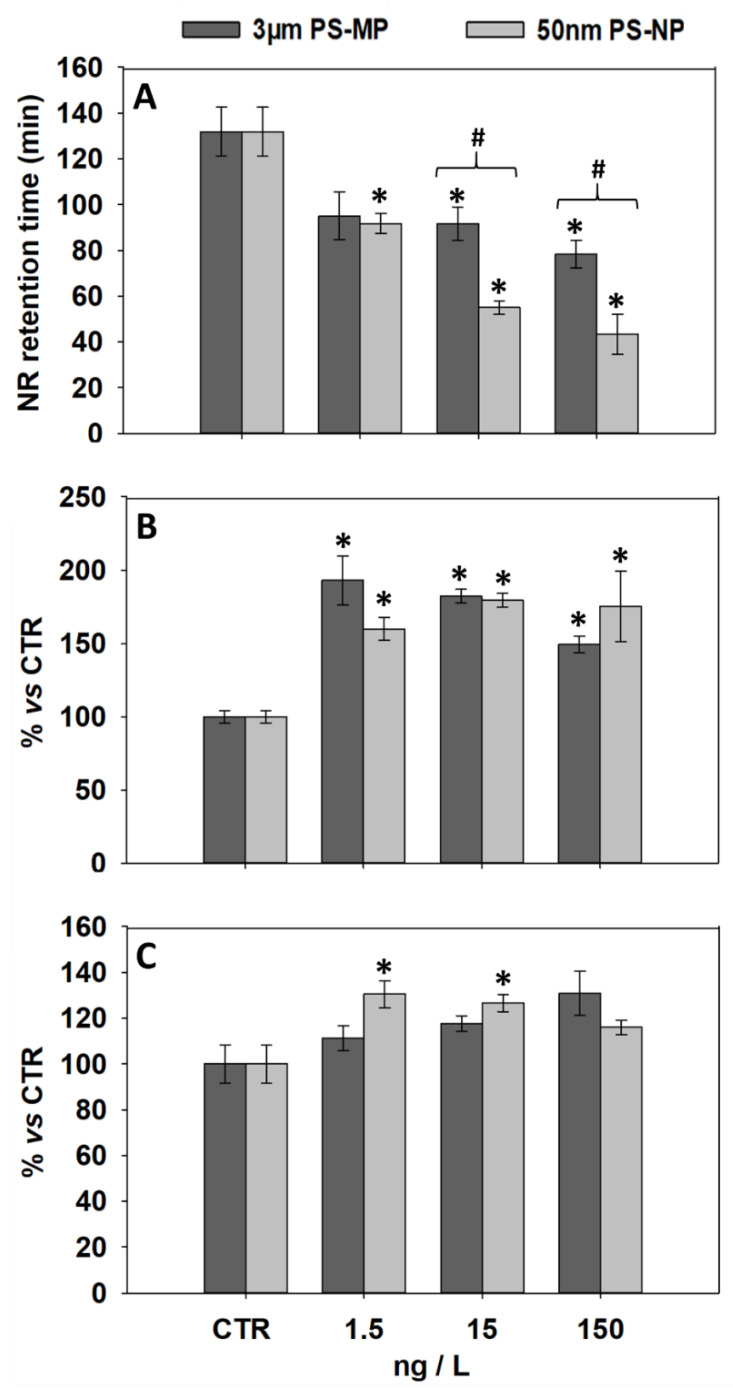
Lysosomal parameters (mean ± SEM, N = 3) analyzed in mussels after 21 days of exposure to polystyrene microplastics (PS-MP) and polystyrene nanoplastics (PS-NP); (**A**), Lysosomal Membrane Stability (LMS) assessed in mussel hemocytes; (**B**), lysosomal content of unsaturated neutral lipids (NL) in mussel digestive gland; (**C**), lysosome to cytoplasm volume ratio (LYS/CYT) in mussel digestive cells. *, significant (*p* < 0.05) differences compared to control samples (CTR); #, significant differences (*p* < 0.05) between PS-MP and PS-NP treatments.

The effects induced by PS-MP and PS-NP on selected lysosomal parameters are shown in Figure 1. With respect to control, both types of particles induced a significant LMS decrease in mussel hemocytes at all tested concentrations, except for 1.5 ng/L PS-MP (Figure 1A). In addition, NRRTs measured at 15 and 150 ng/L PS-NP showed significantly lower levels compared to those observed at the same PS-MP concentrations.

NL levels in the mussel digestive gland showed a significant increase versus control after exposure to all tested concentrations of PS-MP and PS-NP (Figure 1B). LYS/CYT showed no significant change versus control in mussels exposed to all PS-MP treatments, while a significant increase was observed after exposure to 1.5 and 15 ng/L PS-NP (Figure 1C). NL and LYS-CYT levels did not statistically differ between mussels exposed to PS-MP and PS-NP treatments (Figure 1B,C).

### 3.2. Effects of PS-MP and PS-NP on Levels of LF and MDA

**Figure 2:**
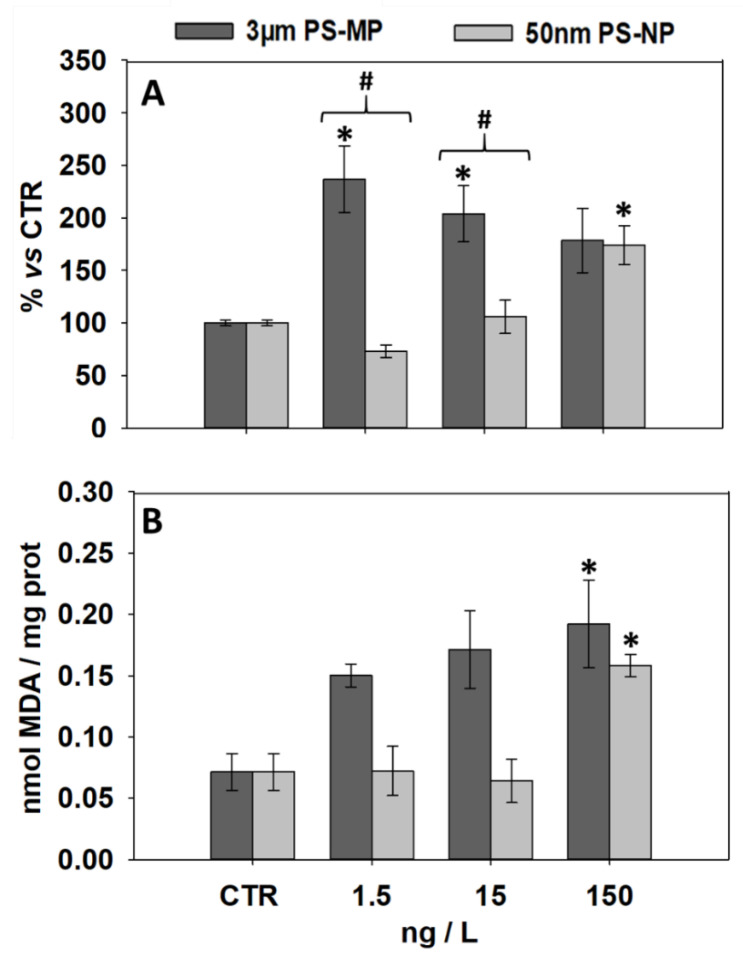
Lipid peroxidation parameters (mean ± SEM, N = 3) analyzed in mussels after 21 days of exposure to PS-MP and PS-NP; (**A**), intra-lysosomal content of lipofuscin (LF) content in the digestive gland; (**B**), malondialdehyde (MDA) content in the digestive gland. *, significant (*p* < 0.05) differences compared to control samples (CTR); #, significant differences (*p* < 0.05) between PS-MP and PS-NP treatments.

The levels of lipid peroxidation products LF and MDA assessed in the digestive gland of mussels exposed to PS-MP and PS-NP are shown in Figure 2. Compared to the control, LF significantly increased at 1.5 and 15 ng/L PS-MP and at 150 ng/L PS-NP (Figure 2A). Mussels exposed to 1.5 and 15 ng/L PS-NP showed significantly lower LF values than observed at the same concentrations of PS-MP. MDA content was up-regulated only by the exposure to 150 ng/L of both PS particle sizes (Figure 2B). Overall, MDA levels did not show significant differences between PS-NP and PS-MP treatments.

### 3.3. Effects of PS-MP and PS-NP on the Activities of GST and CAT

**Figure 3:**
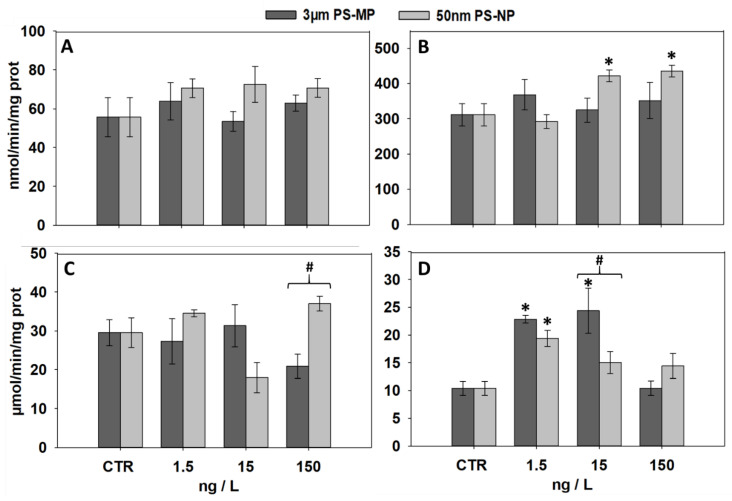
Activity of antioxidant/detoxification enzymes (mean ± SEM, N = 3) analyzed in mussels after 21 days of exposure to PS-MP and PS-NP; (**A**,**B**), glutathione S-transferase (GST) activity in mussel digestive gland (**A**) and gills (**B**); (**C**,**D**), catalase (CAT) activity in mussel digestive gland (**C**) and gills (**D**); *, significant (*p* < 0.05) differences compared to control samples (CTR); #, significant differences (*p* < 0.05) between PS-MP and PS-NP treatments.

The effects induced by PS-MP and PS-NP on the specific activity of the enzymes GST and CAT are displayed in Figure 3. In the digestive gland, no change in GST activity was observed compared to control after exposure to all treatments of both particle sizes (Figure 3A), while a significant increase was induced in gills of mussels exposed to 15 and 150 ng/L PS-NP (Figure 3B). Similarly, CAT activity in the digestive gland showed no change versus control after exposure to all treatments, although levels measured at 150 ng/L PS-MP were significantly lower than those measured at the same concentration of PS-NP (Figure 3C). In gills, a significant CAT activity increase was induced by the exposure to 1.5 ng/L of both particle types and at 15 ng/L PS-MP, the latter showing significantly higher levels compared to the same concentration of PS-NP.

### 3.4. Effects of PS-MP and PS-NP on Immunological and Neurological Parameters

**Figure 4:**
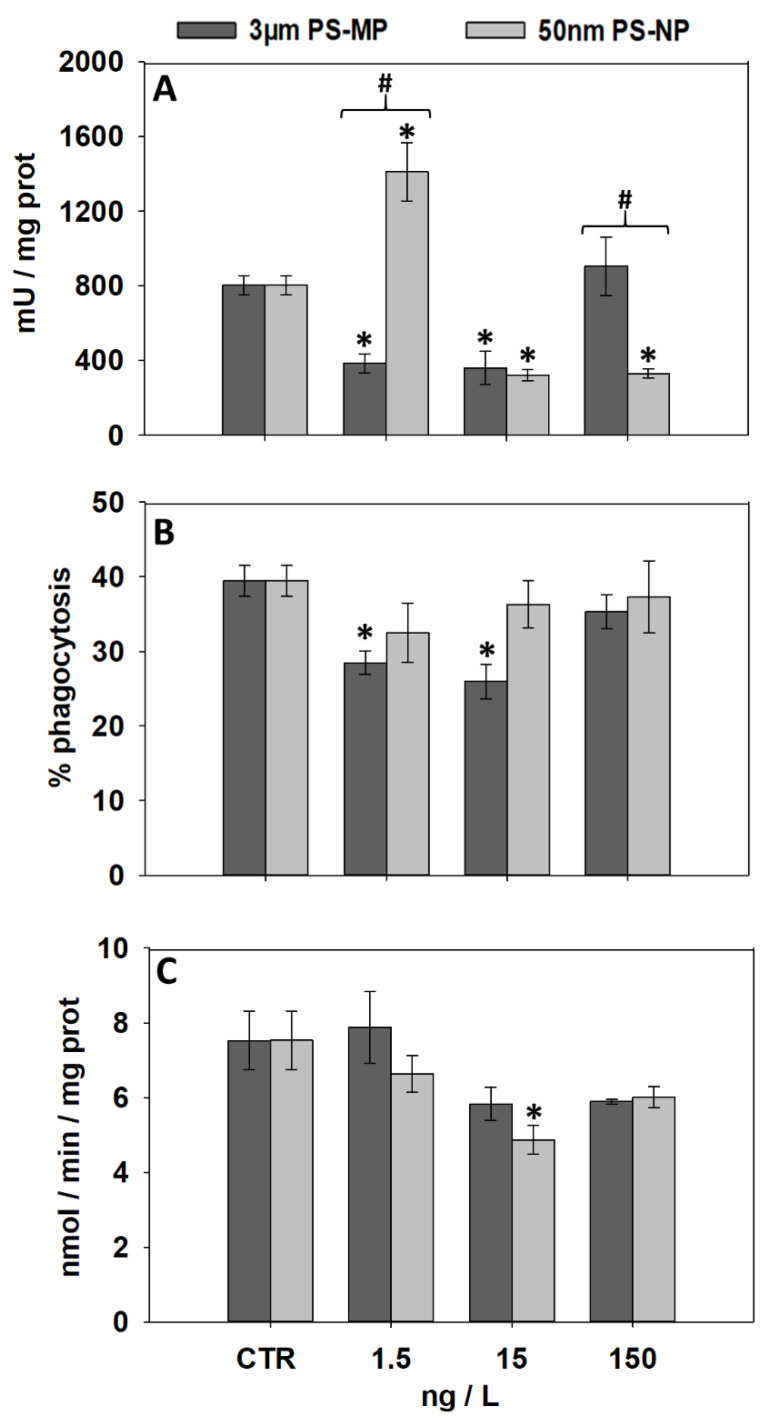
Immunological and neurological parameters (mean ± SEM, N = 3) analyzed in mussels after 21 days of exposure to PS-MP and PS-NP; (**A**), lysozyme activity (LSZ) in the hemolymph serum; (**B**), hemocytes phagocytic activity; (**C**), acetylcholinesterase (AChE) activity in gills. *, significant (*p* < 0.05) differences compared to control samples (CTR); #, significant differences (*p* < 0.05) between PS-MP and PS-NP treatments.

Figure 4 shows the effects induced by increasing PS-MP and PS-NP concentrations on the hemolymph serum lysozyme activity, the hemocyte phagocytosis, and the AChE activity in gills. Compared to controls, a significantly decreased lysozyme activity was observed after mussel exposure to 1.5 and 15 ng/L PS-MP, while PS-NP treatments resulted in a significant increase at 1.5 ng/L followed by a decrease at 15 and 150 ng/L (Figure 4A). Compared to PS-NP, levels of lysozyme activity measured in PS-MP-exposed mussels were significantly lower at 1.5 ng/L and significantly higher at 150 ng/L (Figure 4A).

The phagocytic activity of mussel hemocytes was significantly inhibited compared to the control after the exposure to 1.5 and 15 ng/L PS-MP (Figure 4B); conversely, no effect was observed at any PS-NP treatments and no difference was observed, at any treatment, between PS-MP and PS-NP treatments (Figure 4B). No effect was induced by any PS-MP treatments on the activity of AChE measured in mussel gills (Figure 4C); on the other hand, a significant AChE decrease was induced by PS-NP at 15 ng/L. No significant change was noted between the AChE activity measured in PS-NP- and PS-MP-treated mussels (Figure 4C).

### 3.5. PCA and MES Data Integration

**Figure 5:**
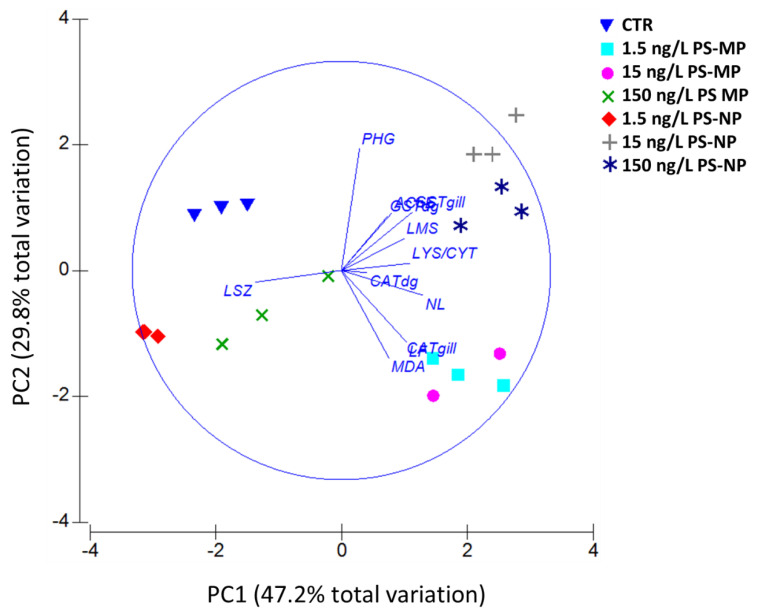
Principal component analysis (PCA). Biplot showing the PCA output including the entire set of biological data obtained in mussels exposed to increasing concentrations of PS-MP and PS-NP for 21 days.

The output of the PCA performed on data from PS-MP and PS-NP treatments is shown in Figure 5. The first two axes accounted for 77% of the total variance. Data from control (CTR) clustered together in the PC1 < 0/PC2 > 0 domain. Data from 1.5 and 15 ng/L PS-MP scored for PC1 > 0/PC2 < 0. Data from 150 ng/L PS-MP and 1.5 ng/L PS-NP were scaled at PC1 < 0/PC2 < 0, while those from 15 and 150 ng/L PS-MP show coordinates > 0 for both PC1 and PC2. According to variable vectors, data from phagocytosis (PHG), LMS, LYS/CYT, GST, and AChE show coordinates in the PC1 > 0/PC2 > 0 domain; NL and LF, as well as MDA and CAT clustered in the PC1 > 0/PC 2 < 0 domain, while lysozyme activity (LSZ) was the only variable clustering at PC1 < 0/PC 2 < 0.

The output of the biomarker data integration performed through the MES is shown in Table 1. The LMS was identified as the guide parameter given its known sensitivity to sub-lethal disturbances and its representativeness of stress-induced health status deterioration in mussels [26,37]. The system did not identify health alterations in controls and 1.5 ng/L MP treatments (HSI = A), while the onset of a low-stress level (HSI = B) was detected at 15 and 150 ng/L MP. Differently, the stress level associated with NP treatments was moderate (HSI = C) at 1.5 and 150 ng/L, and high (HSI = D) at 15 ng/L.

## 4. Discussion

The fragmentation of MP to nano-scaled particles is acknowledged as a real environmental risk, notably concerning the higher potential for < 100 nm plastics to cross biological membranes [4]. In this respect, the present study aimed at comparatively assessing the chronic effects of polystyrene micro and nanoparticles on the cellular and physiological fitness of the Mediterranean mussel *M. galloprovincialis*.

The LMS is the most sensitive biomarker of general stress in mussels, and its reduction is recognized as prognostic of alterations to higher functions, such as growth and/or reproduction [38,39]. In this study, an overall LMS reduction was observed in *M. galloprovincialis* hemocytes after 21 days of exposure to both PS-MP and PS-NP, corroborating recent evidence obtained in bivalves exposed to plastic particles of diverse types and sizes [40]. In addition, data reveal an overall higher lysosomal destabilization triggered by PS-NP compared to PS-MP treatments. Previous reports suggest that these differences might be attributed to a substantially higher lysosomotropic behavior of nano-sized plastics. In fact, mechanistic links were highlighted between LMS alterations in oysters and the intra-lysosomal migration of nanomaterials, including gold, silver, and fullerene nanoparticles (3 to 25 nm) [41]. More recent findings obtained in mammal fibroblasts further indicate that 50-nm PS-NP readily migrates into lysosomes, leading to dysfunctions spanning from the blockage of degradative pathways to a severe increase of the lysosomal membrane permeabilization [42].

The bivalve digestive gland is responsible for key metabolic functions, including food intracellular uptake/digestion and reserve substances storage, among others [43]. The lysosomal swelling in cells surrounding the tubular epithelium of the mussel digestive gland is a known physio-pathological condition induced by stress factors of chemical and physical nature [30,44]. Between PS-NP and PS-MP, only the former induced an LYS/CYT increase in the mussel digestive cells. This may reflect the onset of an NP-mediated stimulation of intra-lysosomal migration pathways, which ultimately led to increased lysosomal volume by altering autophagic processes and enhancing cell vacuole aggregation. In line with this hypothesis, Gaspar et al. [45] recently observed that 50-nm PS particles exhibit a much greater propensity for endo-lysosomal accumulation in oyster (*Crassostrea virginica*) hepatopancreas cells compared to 3-µm PS-MP, indicating the potential for a higher bioreactivity and interaction with the lysosome integrity and functions. NL content measured in the mussel digestive gland showed a significant up-regulation after exposure to both PS particle sizes. These data contrast with the NL unmodified levels observed by Avio et al. [46] following a shorter (7-day) exposure of mussels to PS and polyethylene MP of different sizes. In bivalves, the NL intra-lysosomal accumulation is a known signal of lipidosis associated with either an increased cytosolic lipid content or a decreased fatty acid metabolism [26,47]. The results suggest that the prolonged uptake of “non-nutritious” PS particles may lead to the indirect depletion of energy reservoirs involved in lipid processing pathways, a physio-pathological condition already reported in the liver of mammalian models exposed to PS microparticles [48].

Morphological and functional alterations of the mussel lysosomal compartment are frequently associated with pro-oxidant conditions, which might give rise to the peroxidation of the lipid bilayer composing cell membranes [30,33]. MDA and LF are intermediate and final products of lipid peroxidation, respectively, and their determination in the mussel digestive gland has extensively been validated to ascertain the evolution of ROS-mediated effects of micro/nanoparticles of diverse nature [46,49,50,51]. The 21-d exposure to PS-MP and PS-NP resulted in the up-regulation of MDA and LF content in the mussel digestive gland, indicating that lipid peroxidation phenomena might be induced by PS particles irrespective of their size. However, though MDA followed a relatively similar trend of activation at increasing concentrations of both PS particle sizes, LF displayed higher sensitivity at low PS-MP when compared to PS-NP. LF are the result of the lipid peroxidation residues binding with intracellular food degradation by-products, including oxidatively modified proteins, carbohydrates, and/or metals, and are thus thought to reliably reflect the organisms’ nutritional status [52]. As a conservative strategy, marine mussels tend to down-regulate their filtration rate when exposed to increasing concentrations of plankton-sized plastic items [53]. In this respect, the decreasing LF profile at increasing PS-MP could reflect a progressive reduction of the feeding/filtration rate at increasing PS-NP dosages, which would also explain the partial discrepancy with respect to MDA.

To better evaluate the extent of oxidative stress conditions triggered by PS-MP and PS-NP, the MDA and LF measurements were associated with the analysis of the specific activity of the antioxidant/detoxification enzymes CAT and GST. Despite an up-regulation of lipid peroxidation products, CAT and GST activities were not affected in the mussel digestive gland, suggesting that pro-oxidative damages were not such to induce major perturbations and antioxidant responses in this tissue. On the other hand, both enzymes seem to show a mutually interconnected trend in gills with respect to PS-NP treatments: while a CAT up-regulation was observed at 1.5 ng/L, higher dosages corresponded to a progressively lower CAT activity and a significant GST up-regulation. Such a complementary modulation may provide a reliable clue on the mechanisms underlying the ROS-scavenging network activated in mussel gills in response to plastic nanoparticles, corroborating dose-response pathways observed in response to increasing levels of diverse physico-chemical stressors [54]. PS-MP did not induce GST changes in mussel gills, while CAT showed a typical bell-shaped trend, indicating that the antioxidant response to PS items could remarkably change depending on either the particle size or the analyzed tissue.

One of the most concerning risks of the MP and NP uptake is related to their potential to interfere with innate immune responses, altering the organisms’ defense mechanisms toward microbiological stressors [55,56,57,58]. In bivalves, the hemolymph is the major biological compartment involved in the immune response and recent evidence indicates clear concentration-dependent trends of migration and accumulation of PS particles in this tissue, even after short-term (4-day) exposures [17]. Within the hemolymph, the first line of defense toward host interactions is represented by the extracellular and intracellular degradation mechanisms governed by the hemocytes, including the lysozyme extracellular release and the phagocytosis of non-self material [59,60]. The exposure to PS-MP and PS-NP had an overall inhibiting effect on the lysozyme activity in the mussel hemolymph serum, except for 1.5 ng/L PS-NP, which led to a sharp lysozyme up-regulation. A lysozyme inhibition is a clear signal of alteration of the hemocyte bactericidal competence, which might expose organisms to higher risks for host colonization or infection [61]. Lysozyme alterations have frequently been reported in bivalves following exposure to many classes of chemical and physical stressors (reviewed by Giròn-Perez, 2010). In a previous in vitro investigation, Canesi et al. [59] showed that the lysozyme activity of mussel hemolymph serum is activated within 30 min from exposure to amino-modified polystyrene NP (PS-NH2) but drops to basal levels thereafter. Similarly, an in vivo investigation recently performed by Auguste et al. [24] showed that repeated mussel exposures for 24 h to PS-NH2 resulted in a bell-shaped lysozyme activity in hemolymphs. This suggests that under a PS-NP gradient and following long-term exposures, the lysozyme synthesis and activity might show an initial “host-driven” enhancement followed by a sharp down-regulation, likely resulting from increased catabolic activity in response to the external insult, as reported for other classes of enzymes in mussels [26].

The results showed a general inhibition of the hemocyte phagocytic activity in mussels exposed to PS-MP. A decreased phagocytosis is associated with an impaired fitness of the cell-mediated host responses. In line with our findings, Tang et al. [62] recently reported a decreased phagocytic activity in hemocytes of blood clams (*Tegillarca granosa*) exposed to 0.5–30 µm PS-MP. No down-regulation was however observed in *Mytilus* spp. following sub-chronic (3 h–7 d) exposures to <100-µm PS particles [46,63,64], suggesting that MP-related alterations of immunological processes are functionally expressed in mussels only after relatively long periods of exposure. The direct MP phagocytosis has been documented in mussel hemocytes [63]. Although the adverse effects (directly or indirectly) of MP phagocytosis on the endo-lysosomal system still need to be fully elucidated, it cannot be ruled out that this phenomenon may limit the overall cell phagocytic capacity, thus explaining the decreased zymosan particle phagocytosis observed herein.

Interestingly, the exposure to increasing PS-MP concentrations produced similar U-shaped effects on lysozyme and phagocytic activity, while notably the former showed an opposite trend in response to PS-NP, as previously described. Hormetic responses to plastic particles have been reported in many aquatic species and suggest the modulation of adaptation strategies in the presence of increasing stress stimulus [65,66,67,68]. Although the influence of particle size in shaping these processes remains to be established, the results suggest that MP and NP produce distinct host-driven responses in mussel hemocytes, which in turn may activate different compensatory mechanisms at increasing concentrations.

This study also provides evidence of potential neurotoxicity in mussels exposed to PS-NP, ascribable to a reduction of the AChE specific activity. AChE is involved in the nervous stimulus release and its selective inhibition represents the mechanism of action of many organochlorine pesticides [69]. In aquatic species, the AChE down-regulation is a known consequence of toxicant exposure and may result in alterations of synaptic pathways governing muscle contraction or heart beating [34,70]. Further studies demonstrated that plastic particles of different size affect cholinergic pathways in bivalve mollusks [46,71] and evidence also exist for teleost fish [72,73]. In this respect, the results highlight the need for a better understanding of the mechanisms underlying the anti-cholinesterasic effects of plastic nanoparticles in order to estimate the possible consequences of plastic fragmentation on the neurological fitness of the exposed biota.

The PCA highlighted the spatial clusterization of data from control with respect to treated mussels; however, treatments differing for concentration and/or size of proffered PS particles tended to cluster together based on the measured biological endpoints. This can be observed for 1.5 and 15 ng/L PS-MP, which clustered together depending on oxidative stress parameters LF, MDA, and CAT (in gills). Treatments of 15 and 150 ng/L PS-NP also showed similar clustering based on GST and LMS data, while scores for 150 ng/L PS-MP and 1.5 ng/L PS-NP seem to be mainly attributed to the variations induced by these treatments on the lysozyme activity (LSZ).

The integration of biomarker data in the MES assigned an absent to low stress level (HSI = A − B) to alterations observed in PS-MP exposed mussels, while moderate to high stress (HSI = C − D) was assigned to PS-NP treatments. Overall, this highlights that the 21-day exposure to 1.5, 15, and 150 ng/L PS-NP had an overall higher impact on the mussel physiology compared to PS-MP, indicating that the environmental fragmentation of MP to nano-sized particles may adversely affect the fitness of exposed organisms. As suggested by the calculated AFs and ALs, differences in HSI levels apparently reflect a higher impact of PS-NP on selected general stress biomarkers (notably LMS, chosen as the guide parameter, and LYS/CYT), confirming the role of lysosomal parameters in modulating the fitness of mussels along an increasing stress gradient. Interestingly, the overall stress level exposure decreased from high to moderate at increasing concentrations of PS-NP (i.e., from 15 to 150 ng/L). This apparently reflects the bell-shaped modulation of oxidative stress (i.e., LF) lysosomal and immunological parameters, which might result from the mussel’s ability to moderate its filtration rate in the presence of an increasing external stressor stimulus [53].

## 5. Conclusions

This study demonstrated that the 21-day exposure to PS-MP and PS-NP can affect the fitness of the Mediterranean mussel *M. galloprovincialis* by selectively modulating biochemical, cellular, and physiological processes. PS-NP had an overall greater effect than PS-MP on lysosomal parameters, as LMS and LYS/CYT, suggesting a higher potential for nano-sized plastics to elicit lysosomal dysfunctions associated with the onset of a general stress syndrome. Both particle types induced lipid peroxidation and a bell-shaped activation of the antioxidant enzyme CAT; however, PS-NP induced greater effects on GST and lysozyme activities, while only PS-MP inhibited the hemocyte phagocytosis, suggesting that the PS particle size play a major role in modulating immunological and detoxification pathways in mussels. In addition, only PS-NP affected the AChE activity, accenting the potential for plastic nano-fragments to impair neurological functions in the long term.

Overall, the results highlight that the fragmentation of PS microparticles to nano-scaled fragments might enhance their ability to interfere with lysosomal, neurological, and immunological functions, leading to an exacerbation of the impacts induced on the mussel health conditions. Further efforts are necessary to ascertain the interaction of plastics of different sizes at the cell level, notably for what concerns the ability of nanoplastics to cross biological membranes and interact with key processes involved in cell homeostasis and functions in mussels. The findings obtained herein may support risk assessment models for marine plastic pollution and provide valuable clues for future investigations aimed at ascertaining the cumulative risks associated with the sorption (or leaching) of chemical pollutants/additives on the mechanisms modulating the physiological fitness of marine organisms.

## Figures and Tables

**Table 1:** Output of the Mussel Expert System (MES) assigning a unique health status index (HSI) to mussels exposed for 21 days to increasing concentrations of PS-MP and PS-NP.

Biomarker		1.5 ng/L MP	15 ng/L MP	150 ng/L MP	1.5 ng/L NP	15 ng/L NP	150 ng/L NP
***Cell***							
LMS^GP^	**AF**	NV	0.70 *	0.59 *	0.70 *	0.42 *	0.33 *
	**AL**	NV	-	-	-	--	--
NL	**AF**	1.93 *	1.82 *	1.49 *	1.60 *	1.79 *	1.75 *
	**AL**	+	+	+	+	+	+
LF	**AF**	2.37 *	2.04 *	1.78	0.73	1.06	1.74 *
	**AL**	+	NV	NV	NV	NV	+
MDA	**AF**	2.08	2.38	2.67 *	1.00	0.89	2.19 *
	**AL**	NV	NV	++	NV	NV	++
GST_dg_	**AF**	1.15	0.96	1.13	1.27	1.30	1.27
	**AL**	NV	NV	NV	NV	NV	NV
GST_g_	**AF**	1.18	1.04	1.13	0.94	1.35 *	1.40 *
	**AL**	NV	NV	NV	NV	+	+
CAT_dg_	**AF**	0.93	1.06	0.71	1.17	0.61	1.25
	**AL**	NV	NV	NV	NV	NV	NV
CAT_g_	**AF**	2.19 *	2.24 *	1.00	1.86 *	1.44	1.38
	**AL**	++	++	NV	+	NV	NV
AChE	**AF**	1.05	0.77	0.78	0.88	0.65 *	0.80
	**AL**	NV	NV	NV	NV	-	NV
Lysozyme	**AF**	0.48 *	0.45 *	1.13	1.75 *	0.40	0.41
	**AL**	NV	NV	NV	+	NV	NV
Phagocythosis	**AF**	0.72 *	0.66 *	0.90	0.82	0.92	0.95
	**AL**	NV	NV	NV	NV	NV	NV
***Tissue***							
LYS/CYT	**AF**	1.11	1.18	1.31	1.30 *	1.27 *	1.16
	**AL**	NV	NV	NV	+	+	NV
***Organism***							
Survival	**AF**	1.00	1.00	1.00	1.00	1.00	1.00
	**AL**	NV	NV	NV	NV	NV	NV
**HSI**		**A (Healthy)**	**B (Low stress)**	**B (Low stress)**	**C (Moderate stress)**	**D (High stress)**	**C (Moderate stress)**

LMS—lysosomal membrane stability; NL—neutral lipid content; LF—lipofuscin content; MDA—malondialdehyde content; GST_dg_—glutathione S-transferase activity analyzed in digestive gland; GST_g_—glutathione S-transferase activity analyzed in gills; CAT_dg_—catalase activity analyzed in digestive gland; CAT_g_—catalase activity analyzed in gills; AChE—acetylcholinesterase activity; LYS/CYT—lysosome/cytoplasm volume ratio; GP—guide parameter; HSI—health status index; AF—alteration factor; AL—alteration level. AL thresholds for increasing/bell-shaped biomarkers: “NV” (no variation) = AF < 1.2; “+” = AF > 1.2; “++” = AF > 2.00; AF thresholds for decreasing biomarkers: “NV” = AF > 0.8; “-” = AF < 0.8; “--” = AF < 0.5. “*”, *p* < 0.05 vs. control.

## Supplementary Materials

The following are available online at https://www.mdpi.com/2079-4991/11/3/649/s1, Section S1: Selected polystyrene micro- and nano-sized particles, Table S1: Tissue treatment preliminary to the analysis of enzymatic/biochemical biomarkers.
