# Supplementary material for: A Comparative Assessment of the Chronic Effects of Micro- and Nano-Plastics on the Physiology of the Mediterranean Mussel Mytilus galloprovincialis
Source: Nanomaterials (Basel). 2021 Mar 7;11(3):649. doi: 10.3390/nano11030649 (PMC8001054; doi:10.3390/nano11030649)
Supplement: Supplementary file 1 [file nanomaterials-11-00649-s001.pdf]

Supplementary Material

A Comparative Assessment of the Chronic Effects of Micro- and Nano-Plastics on the Physiology of the Mediterranean Mussel *Mytilus galloprovincialis*

Marco Capolupo *, Paola Valbonesi and Elena Fabbri

Department of Biological, Geological, and Environmental Sciences, University of Bologna, Via Sant' Alberto 163, 48123 Ravenna, Italy

*Corresponding Author: Marco.capolupo2@unibo.it (M.C.)

NUMBER OF PAGES: 3

Section S1 (page 2)

Table S1 (page 3)

Section S1. Selected polystyrene micro- (PS-MP) and nano-sized particles (PS-NP)

Three-micrometer and fifty-nanometer polystyrene beads (3 ± 0.15 μm and 43 ± 4 nm size diameter, respectively) were purchased from Polyscience Inc. (Washington, PA, USA). These particles were selected as a proxy of MP/NP exposure since their density (1.05 g/cm^3) is almost identical to that of artificial seawater (about 1.025–1.035 g/cm^3), so that they should not sink within 48 h from suspension in water. Charge change is unlikely, as the microsphere are neutral and, according to the manufacture's specifications, only covalent interactions should be allowed to bind proteins or nucleic acids. Surfactants were not present or added to the original particle suspension, whose medium was ultrapure water (Polyscience, Inc.). Stock solutions at the suitable concentrations were prepared in sterile distilled water, aliquoted and stored at 4 °C according to the manufacturer's specification. At each MP administration, 1 aliquot of the stock solution was serially diluted in sterile artificial seawater (ASW) to achieve the final treatment concentration in the tanks. Before spiking, lack of aggregation was verified microscopically.

Table S1. Tissue treatment preliminary to the analysis of biochemical biomarkers. In all cases, tissues were homogenized using a Potter glass homogenizer.

Biomarker	Tissue homogenation	Centrifugation	Total protein quantification	Reference
GST, CAT	400–600 mg gill/digestive gland in 5 vol. of 50 mM KPB, pH 7.4 (0.5 mM Na_2EDTA and protein inhibitor at 1 μL/100 mg tissue)	10,200 ×g for 15 min at 4 °C	Folin–Ciocalteu reaction [27]*	[33]*
MDA	1 g digestive gland in 2 vol. of 20 mM Tris-HCl pH 7.4 (0.1% β-mercaptoethanol)	10,400 ×g for 20 min at 4 °C		[32]*
AChE	500 mg gills in 4 vol. 0.1 M sodium-phosphate buffer, pH 7.4	10,200 rpm for 30 mins at 4 °C		[34]*

GST, glutathione S-transferase activity; CAT, Catalase activity; MDA, malondialdehyde content. AChE, acetylcholinesterase activity; KPB, potassium-phosphate buffer; *, citation numbered as in the main text.
